# Acute ischemic stroke with cervical internal carotid artery steno-occlusive lesion: multicenter analysis of endovascular approaches

**DOI:** 10.1186/s12883-021-02393-4

**Published:** 2021-09-17

**Authors:** Luigi Cirillo, Daniele Giuseppe Romano, Gianfranco Vornetti, Giulia Frauenfelder, Chiara Tamburrano, Francesco Taglialatela, Salvatore Isceri, Renato Saponiero, Rosa Napoletano, Mauro Gentile, Michele Romoli, Ciro Princiotta, Luigi Simonetti, Andrea Zini

**Affiliations:** 1 Department of Experimental, Diagnostic and Speciality Medicine, University of Bologna, Bologna, Italy; 2 UOSI Neuroradiologia Ospedale Maggiore CA Pizzardi - IRCCS delle scienze Neurologiche di Bologna, Bologna, Italy; 3 UOC Neuroradiologia AOU S. Giovanni di Dio e Ruggi d’Aragona, Salerno, Italy; 4 UOC Neurologia AOU S. Giovanni di Dio e Ruggi d’Aragona, Salerno, Italy; 5 UOC Neurologia e Rete Stroke metropolitana Ospedale Maggiore CA Pizzardi - IRCCS delle scienze Neurologiche di Bologna, Bologna, Italy

**Keywords:** Acute ischemic stroke, Internal carotid artery occlusion, Tandem lesion, Endovascular treatment.

## Abstract

**Background:**

Occlusion of the internal carotid artery (ICA), whether isolated or in the setting of a tandem lesion (TL) have a poor response to treatment with intravenous thrombolysis. Previous studies ​​have demonstrated the superiority of mechanical thrombectomy in the treatment of acute ischemic stroke (AIS) following large vessel occlusion, compared to standard intravenous fibrinolysis. The aim of our study was to describe endovascular treatment (EVT) in AIS due to isolated ICA occlusion or TL.

**Methods:**

We assessed the association between 90-day outcome and clinical, demographic, imaging, and procedure data in 51 consecutive patients with acute isolated ICA occlusion or TL who underwent EVT. We evaluated baseline NIHSS and mRS, ASPECTS, type of occlusion, stent placement, use of stent retrievers and/or thromboaspiration, duration of the procedure, mTICI, postprocedural therapy and complications.

**Results:**

A favorable 90-day outcome (mRS 0–2) was achieved in 34 patients (67 %) and was significantly associated with the use of dual antiplatelet therapy after the procedure (*p* = 0.008), shorter procedure duration (p = 0.031), TICI 2b-3 (*p* < 0.001) and lack of post-procedural hemorrhagic transformation (*p* = 0.001). Four patients did not survive, resulting in a mortality rate of 8 %.

**Conclusions:**

Our study has shown that EVT in the treatment of AIS due to ICA occlusion is safe, and effective in determining a good functional outcome. ICA stenting led to good angiographic results and therapy with a glycoprotein IIb / IIIa inhibitor immediately after stent release did not result in a greater risk of hemorrhage. The use of post-procedural dual antiplatelet therapy was associated with favorable outcome, without a significant increase in hemorrhagic transformation.

## Background

Ischemic stroke is a devastating condition with a high burden of neurologic disability and death. Timely and effective reperfusion is necessary to salvage the ischemic penumbra and to increase the chances of a favorable clinical outcome [1].

Several factors may influence the response to therapy in acute ischemic stroke (AIS), such as time-to-treatment, size, and location of arterial occlusion. The main reason for the limited efficacy of thrombolysis is the modest rate of early reperfusion among patients with a large-vessel occlusion [2, 3], such as those with internal carotid artery (ICA) involvement. There are two types of AIS due to ICA occlusion that show a poor prognosis for inadequate response to systemic treatment: complete occlusion of ICA due to atheromatous plaque or dissection and tandem lesions (TL), defined as proximal anterior circulation intracranial occlusion and an ipsilateral cervical ICA high-grade stenosis or occlusion [4].

Acute ICA occlusion causes between 6 and 15 % of AIS and is associated with a significant level of morbidity and mortality [5]. For this type of lesion, treatment with standard intravenous thrombolysis alone leads to a good clinical outcome in only 17 % of cases, with a death rate as high as 55 % [6]. Accordingly, TL response rates to intravenous thrombolysis are low and the prognosis is often poor [7–9].

Since 2015, several trials assessed the superiority of mechanical thrombectomy in the treatment of AIS due large vessel occlusion in the anterior circulation over standard medical care [10–13]. Nevertheless, the efficacy of endovascular treatment in TL, as well as the choice of emergent carotid stenting is not yet clearly defined.

In this study, we report a multicenter experience in EVT of AIS due to ICA occlusion, either in isolation or in the setting of a TL.

## Methods

### Patient Selection

Data collection was carried out from May 2018 to March 2020 at the Hub Centers for ischemic stroke in Bologna (Ospedale Maggiore Carlo Alberto Pizzardi) and in Salerno (Ospedali Riuniti San Giovanni di Dio and Ruggi d’Aragona).

All patients who arrived in the two centers with symptoms of AIS, within 6 h of onset, were evaluated with CT angiography and CT perfusion according to a standard stroke protocol. We included in this study all patients (n = 51) with isolated ICA occlusion (both on an atheromatous basis or due to dissection) or TL.

Ethical approval was waived by the review boards of our institutions (Ospedale Maggiore Carlo Alberto Pizzardi and Ospedali Riuniti San Giovanni di Dio and Ruggi d’Aragona) in view of the retrospective nature of the study. All procedures being performed were in accordance with the 1964 Helsinki Declaration and its later amendments and were part of approved integrated care pathways (PDTAI 017 and PDTA 234 resolution no. 321 of June 18, 2018).

### Demographic and clinical data

We collected the following clinical and demographic data: age, sex, National Institute of Health Stroke Scale (NIHSS), pre-treatment modified Rankin Scale (mRS), previous antiplatelet therapy and comorbidities (hypertension, diabetes, hypercholesterolemia, and atrial fibrillation).

### Neuroimaging features

We reviewed admission non-contrast CT, CT-angiography, and CT perfusion. Baseline Alberta Stroke Program Early CT Score (ASPECTS), type of occlusion (isolated ICA occlusion or TL), and cause of occlusion (either atheromatous or secondary to dissection) were evaluated.

### Procedure data

We collected data on stent placement, use of stent-retriever and/or thromboaspiration, use of dual antiplatelet therapy after the procedure, procedure time, angiographic result using the modified Thrombolysis in Cerebral Infarction score (mTICI): mTICI 0 / 2 A = reperfusion of < 50 % of the vascular territory and mTICI 2B / 3 = reperfusion of ≥ 50 % of the vascular territory. For ICA stenting we employed either Wallstent (Boston Scientific, Marlborough, Massachusetts) or Casper stent (Microvention, Terumo, Tustin, California), while for mechanical thrombectomy with retriever we employed Trevo (Stryker, Kalamazoo, Michigan) or Solitair (Medtronic, Dublin, Ireland), and Sofia (Microvention, Terumo, Tustin, California) or Penumbra (Penumbra, Alameda, California) catheters for thromboaspiration. None of the included patients were treated with IV therapy alone.

The choice of post-procedural antiplatelet therapy (single or dual) was established as follows:

• Dual therapy: patients with stent implantation or patients already on ASA therapy before the ischemic event but who nevertheless developed an AIS;
• Single therapy: patients who were not implanted with a stent and who were not on ASA therapy before the ischemic event or patients with an indication for dual therapy who developed hemorrhagic transformation within 12 h of the procedure (while the patient was still on tirofiban infusion and before starting dual antiplatelet therapy);
• No therapy: patients with single therapy indications who developed hemorrhagic transformation within 12 h of the procedure.

### Outcome

A good clinical outcome was defined as an mRS of 0–2 at 90 days (as assessed by an examiner independent of the interventional physician), while an mTICI score of 2b-3 was considered as successful reperfusion. The 90-day mRS score was obtained through patient visit or phone interview. Any procedure-related complications, such as hemorrhagic infarction (HI) or parenchymal hematoma (PH) were classified according to the European Cooperative Acute Stroke Study II (ECASS II) criteria [9]. At discharge NHISS score was regarded as minor stroke (0–4), moderate stroke (5–15), moderate to severe stroke (16–20) and severe stroke (> 20).

### Statistical Analysis

Categorical variables were compared using Fisher’s exact test, while Wilcoxon rank-sum test was computed for continuous variables. At multivariate analysis, odds ratios (OR) and 95 % confidence intervals (95 % CI) were estimated with a logistic regression model. Categorical variables were summarized by absolute and relative (%) frequency, numerical variables were described as median and interquartile range (IQR). All tests were two sided, and *p* < 0.05 was considered significant. Statistical analysis was performed using R version 3.6.1 (The R Foundation for Statistical Computing, Vienna, Austria, 2019).

## Results

Clinical, neuroimaging and procedure data are summarized in Table 1.

**Table 1:** Clinical, neuroimaging and procedure data

Sex (Female)	19 (37.2 %)
Age (years)	68.3 (61.4–76)
Diabetes	16 (31.3 %)
Hyperlipidemia	24 (47.1 %)
Hypertension	36 (70.6 %)
Atrial fibrilation	7 (13.7 %)
ASPECTS	10 (7–10)
9–10	27 (52.9 %)
8 or less	24 (47.1 %)
Occlusion type	
Atherosclerotic	38 (74.5 %)
Dissection	13 (25.5 %)
Tandem lesion	42 (82.3 %)
Preprocedural therapy	21 (41.1 %)
Intraprocedural therapy	18 (35.3 %)
Postprocedural therapy	
ASA	15 (29.4 %)
ASA + Clopidogrel	33 (64.7 %)
Heparin/none	3 (5.9 %)
Hemorragic transformation	10 (19.6 %)
Procedure time (minutes)	69 (46–90)
Extracranial technique	
PTA	18 (35.3 %)
Stent	6 (11.8 %)
PTA + Stent	15 (29.4 %)
None/other	12 (23.5 %)
Intracranial technique	
ADAPT	28 (54.9 %)
Solumbra	11 (21.6 %)
Retriever	2 (3.9 %)
None	10 (19.6 %)
Stent placement	21 (41.1 %)

Categorical variables are expressed as absolute and relative (%), continuous variables as median and interquartile range (IQR).

*ASPECTS* Alberta stroke program early CT score; *ASA* acetylsalicylic acid; *PTA* percutaneous transluminal angioplasty.

### Study Population

Between May 2018 and March 2020, 51 consecutive patients (63 % males, median age 68 years) with AIS were admitted to our two institutions within 6 h of the onset of symptoms and underwent EVT. Of these, 9 patients (17.6 %) had complete occlusion of the ICA, and 42 (82.4 %) had a TL. Thirty-eight patients (74.5 %) were diagnosed with an atheromatous lesion and 13 (25.5 %) with a dissection of the cervical ICA. Baseline ASPECTS was 9–10 in 27 patients (52.9 %) and ≤ 8 in 24 (47.1 %). Median baseline NIHSS was 12 (IQR = 4.0–20.0). Twenty-one patients (41.2 %) were on antiplatelet therapy (ASA or ASA + clopidogrel) prior to the stroke event.

### Clinical and Angiographic Outcomes

At 3 months a favorable outcome (mRS 0–2) was achieved in 34 patients (66.7 %), 13 patients (25 %) had an mRS > 2 and 4 patients did not survive, resulting in a mortality rate of 8 %. TICI 2B-3 (successful intracranial recanalization) was achieved in 42 patients (82.4 %), TICI 0-2 A in 9 (17.6 %).

A stent was deployed in 21 patients (41.2 %) and 95 % of these had a post-treatment TICI 2B-3, while among patients who did not undergo stent placement 73 % achieved successful recanalization (*p* = 0.064).

Ten patients (19.6 %) experienced post-procedural hemorrhagic transformations, in particular, the rate of post-interventional parenchymal hematoma was 1.9 % for PH1 and 1.9 % for PH2 while the rate of post-interventional petechial hemorrhages was 9.8 % for HI1 and 5.8 % for HI2, in the absence of symptomatic bleeding.

The comparison of patients with successful and unsuccessful intracranial recanalization is reported in Table 2.

**Table 2:** Comparison of patients with successful and unsuccessful intracranial recanalization

Variable	Total *n* = 51	TICI 0-2 A *n* = 9	TICI 2B-3 *n* = 42	p
Sex				1
Male	32	6 (66.6 %)	26 (61.9 %)	
Female	19	3 (33.3 %)	16 (38.1 %)	
Age (years)	68.3 (61.4–76)	72 (63–69.5)	67.9 (58.4–77.2)	0.692
Diabetes				0.436
Yes	16	4 (44.4 %)	12 (28.6 %)	
No	35	5 (55.5 %)	30 (71.4 %)	
Hyperlipidemia				1
Yes	24	4 (44.4 %)	20 (47.6 %)	
No	27	5 (55.5 %)	22 (52.4 %)	
Hypertension				0.709
Yes	36	7 (77.7 %)	29 (69.0 %)	
No	15	2 (22.2 %)	13 (31.0 %)	
Atrial fibrilation				0.095
Yes	7	3 (33.3 %)	4 (9.5 %)	
No	44	6 (66.6 %)	38 (90.5 %)	
ASPECTS				0.718
9–10	27	4 (44.4 %)	23 (54.8 %)	
8 or less	24	5 (55.5 %)	19 (45.2 %)	
Occlusion type				0.417
Atherosclerotic	38	8 (88.8 %)	30 (71.4 %)	
Dissection	13	1 (11.1 %)	12 (28.6 %)	
Occlusion location				1
Tandem	42	8 (88.8 %)	34 (81.0 %)	
Extracranial	9	1 (11.1 %)	8 (19.0 %)	
Preprocedural therapy				0.720
Yes	21	3 (33.3 %)	18 (42.9 %)	
No	30	6 (66.6 %)	24 (57.1 %)	
Intraprocedural therapy				0.134
Yes	18	1 (11.1 %)	17 (40.5 %)	
No	33	8 (88.8 %)	25 (59.5 %)	
Postprocedural therapy				0.001
ASA	15	3 (33.3 %)	12 (28.6 %)	
ASA + Clopidogrel	33	3 (33.3 %)	30 (71.4 %)	
Heparin/none	3	3 (33.3 %)	0 (0 %)	
Hemorrhagic transformation				0.009
Yes	10	5 (55.5 %)	5 (11.9 %)	
No	41	4 (44.4 %)	37 (88.1 %)	
Procedure time (minutes)	69 (46–90)	70 (55–90)	68.5 (45.5–90)	0.683
Extracranial technique				0.081
PTA	18	4 (44.4 %)	14 (33.3 %)	
Stent	6	1 (11.1 %)	5 (11.9 %)	
PTA + Stent	15	0 (0 %)	15 (35.7 %)	
None/other	12	4 (44.4 %)	8 (19.0 %)	
Intracranial technique				0.791
ADAPT	28	5 (55.5 %)	23 (54.8 %)	
Solumbra	11	0 (0 %)	2 (4.8 %)	
Retriever	2	3 (33.3 %)	8 (19.0 %)	
None	10	1 (11.1 %)	9 (21.4 %)	
Stent				0.064
Yes	21	1 (11.1 %)	20 (47.6 %)	
No	30	8 (88.8 %)	22 (52.4 %)	

Categorical variables are expressed as absolute and relative (%), continuous variables as median and interquartile range (IQR).

*TICI* thrombolysis in cerebral infarction; *ASPECTS* Alberta stroke program early CT score; *ASA* acetylsalicylic acid; *PTA* percutaneous transluminal angioplasty.

### Management of ICA occlusion and TL in emergency

Antiplatelet therapy was administered in 18 patients (35.3 %), aspirin in 6 (11.8 %) and GpIIb/IIIa inhibitors in 11 (21.6 %), only 1 patient (1.9 %) received heparin (2000 IU) during the procedure.

A stent was employed in 21 patients (41.2 %). Among patients who underwent intracranial mechanical thrombectomy (80.4 %), ADAPT was employed in 28 (54.9 %), Solumbra in 11 (21.6 %) and retriever in 2 (3.9 %). The median procedure time was 69 min (IQR, 46–90 min).

### Factors Influencing Outcome and Recanalization

The comparison of patients with favorable and unfavorable outcome at 3 months of follow-up is shown in Table 3.

**Table 3:** Comparison of patients with 90-day mRS 0–2 and mRS > 2

Variable	Total *n* = 51	mRS 0–2 *n* = 34	mRS > 2 *n* = 17	p
Sex				0.365
Male	32	23 (67.6 %)	9 (52.9 %)	
Female	19	11 (32.4 %)	8 (47.1 %)	
Age (years)	68.3 (61.4–76)	67.6 (55.6–74.5)	69 (63.1–77)	0.374
TICI				< 0.001
0-2 A	9	0 (0 %)	9 (52.9 %)	
2B-3	42	34 (100 %)	8 (47.1 %)	
Diabetes				0.345
Yes	16	9 (26.5 %)	7 (41.2 %)	
No	35	25 (73.5 %)	10 (58.8 %)	
Hyperlipidemia				0.569
Yes	24	15 (44.1 %)	9 (52.9 %)	
No	27	19 (55.9 %)	8 (47.1 %)	
Hypertension				0.745
Yes	36	23 (67.7 %)	13 (76.5 %)	
No	15	11 (32.3 %)	4 (23.5 %)	
Atrial fibrilation				0.673
Yes	7	4 (11.8 %)	3 (17.6 %)	
No	44	30 (88.2 %)	14 (82.3 %)	
ASPECTS				0.254
9–10	27	20 (58.9 %)	7 (41.2 %)	
8 or less	24	14 (41.2 %)	10 (58.8 %)	
Occlusion type				1
Atherosclerotic	38	25 (73.5 %)	13 (76.5 %)	
Dissection	13	9 (26.5 %)	4 (23.5 %)	
Occlusion location				0.241
Tandem	42	26 (76.5 %)	16 (94.1 %)	
Extracranial	9	8 (23.5 %)	1 (5.9 %)	
Preprocedural therapy				0.081
Yes	21	17 (50.0 %)	4 (23.5 %)	
No	30	17 (50.0 %)	13 (76.5 %)	
Intraprocedural therapy				0.757
Yes	18	13 (38.2 %)	5 (26.5 %)	
No	33	21 (61.8 %)	12 (70.6 %)	
Postprocedural therapy				0.008
ASA	15	8 (23.5 %)	7 (41.2 %)	
ASA + Clopidogrel	33	26 (76.5 %)	7 (41.2 %)	
Heparin/none	3	0 (0 %)	3 (17.6 %)	
Hemorrhagic transformation				0.001
Yes	10	2 (5.9 %)	8 (47.1 %)	
No	41	32 (94.1 %)	9 (52.9 %)	
Procedure time (minutes)	69 (46–90)	60 (45–76.8)	86 (55–119)	0.031
Extracranial technique				0.597
PTA	18	11 (32.3 %)	7 (41.2 %)	
Stent	6	4 (11.8 %)	2 (11.8 %)	
PTA + Stent	15	12 (35.3 %)	3 (17.6 %)	
None/other	12	7 (20.6 %)	5 (26.5 %)	
Intracranial technique				0.098
ADAPT	28	20 (58.8 %)	8 (47.1 %)	
Solumbra	11	4 (11.8 %)	7 (41.2 %)	
Retriever	2	2 (5.9 %)	0 (0 %)	
None	10	8 (23.5 %)	2 (11.8 %)	
Stent				0.366
Yes	21	16 (47.1 %)	5 (26.5 %)	
No	30	18 (52.9 %)	12 (70.6 %)	

Categorical variables are expressed as absolute and relative (%), continuous variables as median and interquartile range (IQR).

*mRS* Modified Rankin Scale; *TICI* thrombolysis in cerebral infarction; *ASPECTS* Alberta stroke program early CT score; *ASA* acetylsalicylic acid; *PTA* percutaneous transluminal angioplasty.

Factors significantly associated with a favorable 90-day outcome were:

- the use of dual antiplatelet therapy after the procedure: 78.8 % of patients who received ASA + clopidogrel had an mRS of 0–2 at 90 days compared to 53.3 % of those who received only ASA (*p* = 0.008).
- A shorter procedure time: median procedure time was 60 min in patients with favorable outcome compared to 86 min in patients with 90-day mRS > 2 (*p* = 0.031).
- lack of post-procedural hemorrhagic transformation: only 2 patients (20 %) among those who had this complication (even though asymptomatic) reached an mRS of 0–2 at 90 days (vs. 78 % of those without hemorrhagic transformation, *p* = 0.001).

Notably, patients with favorable outcome were those who had a satisfactory degree of revascularization. In fact, 81 % of patients with a TICI of 2b-3 had a mRS at 90 days of 0–2, while in the group with a TICI of 0-2a none had a 90-day mRS of 0–2 (p < 0.001). At multivariate analysis, a TICI of 2b-3 was the strongest predictor of favorable outcome (Table 4). Furthermore, this factor affects the mortality rate, which was 2 % in patients with TICI 2b-3 and 33 % in those with TICI 0-2a (p = 0.015).

**Table 4:** Multivariate model for mRS 0–2 vs. mRS > 2 by TICI score, postprocedural therapy and hemorrhagic transformation

	OR	95 % CI	*p*-value
TICI			< 0.001
0-2 A	Ref.	Ref.	
2B-3	1.76	1.32–2.34	
Postprocedural therapy			0.040
ASA/heparin/none	Ref.	Ref.	
ASA + Clopidogrel	1.25	1.02–1.54	
Hemorrhagic transformation			0.008
Yes	Ref.	Ref.	
No	1.45	1.11–1.88	

*mRS* Modified Rankin Scale; *TICI* thrombolysis in cerebral infarction; *OR* odds ratio; *CI* confidence interval; *ASA* acetylsalicylic acid

While the presence of diabetes was not significantly associated with the clinical outcome, 56 % of diabetic patients had an mRS of 0–2 at 3 months, compared to 71 % of those without diabetes. In addition, diabetes also affects the mortality rate (19 % in patients with diabetes vs. 3 % in patients without diabetes, p = 0.085). When considering the association between 90-days mRS and the presence of multiple risk factors, 77.8 % of patients with no risk factors achieved a good clinical outcome compared to 72.7 % among those with one risk factor, 68.2 % among those with two risk factors and 55.6 % among those with three or more risk factors. Nonetheless, this association failed to reach statistical significance (*p* = 0.14).

We did not detect a significant association between clinical outcome and the type of occlusion: a 90-day mRS of 0–2 was reached by 66 % of patients presenting with atheromatous occlusions and by 69 % of those with dissection (*p* = 1). Additionally, we did not find a significant correlation between the degree of severity on the NIHSS scale and the mRS at 3 months.

Good recanalization (TICI 2B-3) was achieved in 95 % of patients who underwent stent placement. Among patients who were given dual antiplatelet therapy subacute hemorrhagic transformation was observed in 17 %, compared to 21 % in patients who did not receive dual antiplatelet therapy.

## Discussion

Timely and effective reperfusion is necessary to reverse the ischemic penumbra and to increase the chance of a favorable clinical outcome [1]. In patients with AIS due to ICA occlusion, recanalization rates after IV therapy alone range from 4.4 to 12.5 % [14]. The reported recanalization rates of IV thrombolysis remained low (10–15 %) even among patients treated within 3 h of stroke onset [15]. The Internal Carotid ARtery Occlusion study (ICARO) showed a higher favorable outcome in patients treated with IV thrombolysis (28.9 %) compared to controls (20.6 %), but an increase in mortality and intracranial bleeding was also observed [16]. Previously published studies suggested that endovascular methods, particularly stenting in extracranial occlusions, achieve better recanalization, higher favorable outcome rates, and lower death rates than IV therapy alone in patients with AIS resulting from occlusion of the ICA [17, 18]. Acute treatment for ischemic stroke has been rapidly evolving over the past 5 years resulting in a dramatic improvement of functional outcome after ischemic stroke in selected patients [19].

In our study the use of a post-procedural dual antiplatelet therapy was significantly associated with the outcome at 3 months (p = 0.008): 78.8 % of patients who underwent this treatment had a 90-day mRS of 0–2 compared to 53.3 % of those who used only one antiplatelet agent or heparin. The association between dual antiplatelet therapy with 90-day mRS may be influenced by indication bias, since it was highly dependent on stent placement, prior ASA use and lack of post-procedural hemorrhagic transformation. Nevertheless, the multivariate model, which included post-procedural therapy as well as TICI score and hemorrhagic transformation among the explanatory variables, showed that dual antiplatelet therapy remained significantly associated with good clinical outcome (p = 0.04, Table 4).

EVT can cause endothelial damage resulting in vessel stenosis, dissections, and reocclusions [20, 21]. Antiplatelet agents might prevent thrombus formation and vessel reocclusion in damaged vessel. Furthermore, administration of antiplatelet therpay is required to prevent reocclusion of stents [22, 23]. However, previous studies showed an increase in bleeding complications in patients undergoing bridging thrombolysis who receive additional antiplatelet therapy during endovascular intervention: the ARTIS trial was stopped early because of an increased rate of symptomatic ICH (sICH) in the patient group in which infusion of 300 mg of ASA was started within 90 min of intravenous thrombolysis with alteplase, with no improvement in outcome [24].

A previous study did not detect an increase in rates of sICH, asymptomatic ICH (aICH) or any bleeding complications, neither in patients receiving ASA acutely nor in patients pretreated with antiplatelet agents [25], in particular sICH (5.6 % without ASA vs. 6.1 % with ASA) and aICH (20 % without ASA vs. 18.8 % with ASA). These data are in agreement with our results: we found hemorrhagic transformation in 19 % of patients treated with antiplatelet agents after EVT (when including both single and dual anti-aggregation). Furthermore, none of these patients developed sICH, and only one patient, who died before the follow-up at 3 months, had parenchymal hematoma type 2 (PH2). Therefore, based on our experience, antiplatelet therapy after EVT is appropriate and safe. No comparison was possible between patients who received antiplatelet therapy and those who did not, since antiplatelet therapy was administered to all but 3 patients (2 were given LMWH and had no bleeding, one was not given any therapy and had HI2 hemorrhagic transformation).

We found similar results when evaluating the association of post-procedural hemorrage with clinical and angiographic outcome. In fact, 78 % of patients without hemorrhagic transformation had an mRS of 0–2 at 3 months, compared to 20 % of those with post-procedural hemorrhage (p = 0.001). Similarly, 90 % of patients without hemorrhagic transformation had a TICI score 2B-3 compared to 50 % of those who presented with post-procedural hemorrhage (p = 0.009).

A significant association is observed between 90-day mRS and the degree of vascularization obtained at the end of the treatment defined as successful intracranial recanalization (TICI 2B-3): 81 % of patients who had a TICI 2B-3 had a 90-day MRS of 0–2 while none of the patients with a TICI of 0-2 A had a 90-day mRS of 0–2 (p < 0.001). Furthermore, this factor affects the mortality rate, which was 2 % in the first group and 33 % in the second group (p = 0.015). This data is in accordance with a previous study which found a correlation between recanalization and outcome in acute ischemic stroke: recanalization is strongly associated with improved functional outcome and reduced mortality [22, 26].

In our study, we analyzed both patients with single ICA occlusion and patients who presented with TL, who underwent EVT. At 3 months a favorable outcome was achieved in 34 patients (66.6 %), while an mRS > 2 was found in 13 patients (25.4 %), with a mortality rate of 8 %. Our data are in accordance with a previous study, which found that the occurrence of recanalization is associated with a 4- to 5-fold increase in the odds of good final functional outcome and a 4- to 5-fold reduction in the odds of death [22].

In all 4 cases of death, the pre-treatment NHISS was higher than 12. Importantly, early NIHSS scores has a strong prognostic value for long-term functional outcome after stroke [27, 28], however the strong correlation between NIHSS and mRS scores does not ensure that the NIHSS is a valid surrogate endpoint [29]. In fact, we did not find a significant association between the degree of severity on the NIHSS scale and mRS at 3 months.

Emergency carotid artery stent placement is expected to reopen an extracranial ICA occlusion with increase of cerebral blood flow in the affected hemisphere. Also, early flow restoration across the tandem lesion in the MCA and/or distal ICA after extracranial ICA stent placement would reverse the ischemic process by stopping the expansion of the ischemic core into the penumbra. The use of stents certainly allows immediate flow restoration but also increases the technical complexity of the procedure. TICI 2B-3 was reported in 95 % of patients in which a stent was deployed compared to 73 % among those who did not undergo stenting (p = 0.064). Stents were used both on isolated ICA lesion and in TL, with a TICI 2B-3 of 100 % in the first group and 94 % in the second group. Therefore, stent placement led to a good angiographic result also in the TL group, even though these patients have a less favorable prognosis.

In our study, antiplatelet therapy with a glycoprotein IIb / IIIa inhibitor immediately after stent release was used in 18 patients (35.3 %). No acute stent thrombosis was observed, while the occurrence of hemorrhagic transformation was 17 % in patients who received antiplatelet therapy and 21 % in patients in whom it was not administered (p = 0.729). Therefore, in our series this therapy was not associated with a greater risk of hemorrhagic transformation. Pre-treatment antiplatelet therapy was used in 21 patients (41.2 %) and 81 % of them had a 90-day mRS 0–2, compared to 57 % among those who did not use antiplatelet therapy (p = 0.081). This result is in accordance with a previous stuty who showed that prior use of antiplatelet therapy improves successful recanalization rate and does not increase the risk of intracranial bleeding in patients affected by AIS due to LVO and treated with EVT [30]. In patients who received pre-treatment antiplatelet therapy, a TICI score of 2B-3 was found in 86 %, which was slightly higher than that found in patients in whom pre-treatment antiplatelet therapy was not administered (80 %).

Among the comorbidities we have considered (diabetes, hypercholesterolemia, hypertension and atrial fibrillation) only atrial fibrillation showed a correlation with worse outcome, with a trend towards significance. In fact, only 57.1 % of patients with atrial fibrillation had a TICI score 2B-3 compared to 86 % of those without it (p = 0.095). Furthermore, considering the Outcome of mRS < 1 at 90 days, the presence of diabetes showed a trend towards significance. In fact, in patients not suffering from diabetes, 54.3 % had an mRS < 1 while in the group of diabetic patients this value dropped to 25 % (p = 0.07). This data ara in agreement with a previous study, which showed a worse functional outcome for diabetic patients with AIS [31]. There is, however, a paucity of studies that assessed the relationship between type 2 diabetes and clinical outcome in AIS [32–34]. It is also relevant to note that 3 out of 4 patients who died before the 3-month follow-up belonged to the group of diabetic patients. Less impactful, but still worth noting, is the fact that a TICI score of 2B-3 is found in 86 % of patients without diabetes and in 75 % of those affected by this disease; however, this difference did not reach statistical significance (p = 0.43). These data can be supported by the observation that diabetes, in addition to being a systemic pathology that affects the patient’s performance, is also related to greater arteriosclerosis and stiffness of the vessels, which significantly affect endovascular technical difficulties.

There were no serious procedure-related complications in our study, such as dissection or vessel rupture. Ten patients (19.6 %) experienced post-procedural hemorrhagic transformation (all asymptomatic), without a significant increase in mortality.

Similarly to what was shown in a previous study [35] we observed a lower mortality rate in our series (8 %) compared to Nedeltchev et al. (20 %) [36]. We hypothesize that the use of new devices and technical advances that have occurred since the time of the study by Nedeltchev et al., such as mechanical clot disruption and mechanical thrombectomy devices, most likely contributed to this difference [35].

Several EVT approaches have been described in the literature, including intra-arterial thrombolysis, microwire clot disruption, angioplasty, thromboaspiration, stenting and thrombectomy with stent retrievers [18]. A previous study showed that emergency stenting in extracranial ICA occlusions achieves higher recanalization rates, better outcome and lower mortality [17], but no final recommendations about the technical procedure are currently available. The optimal approach might depend on the nature of the vessel occlusion, and the time to treatment should be as short as possible [14].

### Limitations

The present study had several limitations. First, it was observational retrospective, so it was prone to selection and other biases. Second, we used multiple different devices for the treatment of tandem occlusions, making it difficult to determine the effect of different thrombectomy techniques. Third, we did not have a control group with which to compare outcome rates. Last, since the main focus of this study is the treatment technique, we did not analyze perfusion imaging to quantify the ischemic area before revascularization.

## Conclusions

Our study has shown that EVT in the treatment of AIS due to ICA occlusion (both on an atheromatous basis and due to dissection) is feasible, safe, and effective in determining a good functional outcome at 90 days; In our study the mortality rate was 8 %, which is similar to the one reported in recent studies [35, 37].

In our experience, stent deployment has proved to be particularly safe and has led to good angiographic results, additionally therapy with a glycoprotein IIb / IIIa inhibitor immediately after stent release did not result in a greater risk of hemorrhage. The use of post-procedural dual antiplatelet therapy was associated with favorable outcome at 3 months, without a significant increase in hemorrhagic transformation.

## Data Availability

The datasets used and/or analysed during the current study are available from the corresponding author on reasonable request.

## Abbreviations

AIS: Acute ischemic stroke
ICA: Internal carotid artery
IV: Intravenous
EVT: Endovascular treatment
mRS: Modified Rankin Scale
mTICI: Modified Thrombolysis In Cerebral Infarction
TL: Tandem lesion
ASPECTS: Alberta stroke program early CT score
ASA: Acetylsalicylic acid

## References

[1] Labeyrie MA, Turc G, Hess A, Hervo P, Mas JL, Meder JF (2012). Diffusion lesion reversal after thrombolysis: A MR correlate of early neurological improvement. Stroke.

[2] del Zoppo GJ, Poeck K, Pessin MS, Wolpert SM, Furlan AJ, Ferbert A (1992). Recombinant tissue plasminogen activator in acute thrombotic and embolic stroke. Ann Neurol.

[3] Bhatia R, Hill MD, Shobha N, Menon B, Bal S, Kochar P (2010). Low rates of acute recanalization with intravenous recombinant tissue plasminogen activator in ischemic stroke: Real-world experience and a call for action. Stroke.

[4] Zhu F, Bracard S, Anxionnat R, Derelle A-L, Tonnelet R, Liao L (2019). Impact of Emergent Cervical Carotid Stenting in Tandem Occlusion Strokes Treated by Thrombectomy: A Review of the TITAN Collaboration. Front Neurol.

[5] Rubiera M, Ribo M, Delgado-Mederos R, Santamarina E, Delgado P, Montaner J (2006). Tandem internal carotid artery/middle cerebral artery occlusion: An independent predictor of poor outcome after systemic thrombolysis. Stroke.

[6] Papanagiotou P, Roth C, Walter S, Behnke S, Grunwald IQ, Viera J (2011). Carotid artery stenting in acute stroke. J Am Coll Cardiol.

[7] Blassiau A, Gawlitza M, Manceau PF, Bakchine S, Serre I, Soize S (2018). Mechanical thrombectomy for tandem occlusions of the internal carotid artery-results of a conservative approach for the extracranial lesion. Front Neurol.

[8] Meves SH, Muhs A, Federlein J, Büttner T, Przuntek H, Postert T (2002). Recanalization of acute symptomatic occlusions of the internal carotid artery. J Neurol.

[9] Adams HP, Bendixen BH, Leira E, Chang KC, Davis PH, Woolson RF (1999). Antithrombotic treatment of ischemic stroke among patients with occlusion or severe stenosis of the internal carotid artery: A report of the Trial of Org 10172 in Acute Stroke Treatment (TOAST). Neurology.

[10] Goyal M, Demchuk AM, Menon BK, Eesa M, Rempel JL, Thornton J (2015). Randomized Assessment of Rapid Endovascular Treatment of Ischemic Stroke. N Engl J Med.

[11] Berkhemer OA, Fransen PSS, Beumer D, van den Berg LA, Lingsma HF, Yoo AJ (2015). A Randomized Trial of Intraarterial Treatment for Acute Ischemic Stroke. N Engl J Med.

[12] Saver JL, Goyal M, Bonafe A, Diener H-C, Levy EI, Pereira VM (2015). Stent-Retriever Thrombectomy after Intravenous t-PA vs. t-PA Alone in Stroke. N Engl J Med.

[13] Jovin TG, Chamorro A, Cobo E, de Miquel MA, Molina CA, Rovira A (2015). Thrombectomy within 8 Hours after Symptom Onset in Ischemic Stroke. N Engl J Med.

[14] Li W, Yin Q, Xu G, Liu X (2016). Treatment Strategies for Acute Ischemic Stroke Caused by Carotid Artery Occlusion. Interv Neurol.

[15] Mori E, Yoneda Y, Tabuchi M, Yoshida T, Ohkawa S, Ohsumi Y (1992). Intravenous recombinant tissue plasminogen activator in acute carotid artery territory stroke. Neurology.

[16] Paciaroni M, Balucani C, Agnelli G, Caso V, Silvestrelli G, Grotta JC (2012). Systemic Thrombolysis in Patients With Acute Ischemic Stroke and Internal Carotid ARtery Occlusion. Stroke.

[17] Kappelhof M, Marquering HA, Berkhemer OA, Majoie CBLM (2015). Intra-arterial treatment of patients with acute ischemic stroke and internal carotid artery occlusion: A literature review. J Neurointerv Surg.

[18] Mokin M, Kass-Hout T, Kass-Hout O, Dumont TM, Kan P, Snyder KV (2012). Intravenous thrombolysis and endovascular therapy for acute ischemic stroke with internal carotid artery occlusion: A systematic review of clinical outcomes. Stroke.

[19] Chalos V, van der Ende NAM, Lingsma HF, Mulder MJHL, Venema E, Dijkland SA (2020). National Institutes of Health Stroke Scale: An Alternative Primary Outcome Measure for Trials of Acute Treatment for Ischemic Stroke. Stroke.

[20] Kurre W, Pérez MA, Horvath D, Schmid E, Bäzner H, Henkes H (2013). Does mechanical thrombectomy in acute embolic stroke have long-term side effects on intracranial vessels? An angiographic follow-up study. Cardiovasc Intervent Radiol.

[21] Almekhlafi MA, Davalos A, Bonafe A, Chapot R, Gralla J, Pereira VM (2014). Impact of age and baseline NIHSS scores on clinical outcomes in the mechanical thrombectomy using solitaire FR in acute ischemic stroke study. Am J Neuroradiol.

[22] Enomoto Y, Yoshimura S (2013). Antiplatelet Therapy for Carotid Artery Stenting. Interv Neurol.

[23] Chaturvedi S, Yadav JS (2006). The role of antiplatelet therapy in carotid stenting for ischemic stroke prevention. Stroke.

[24] Zinkstok SM, Roos YB (2012). Early administration of aspirin in patients treated with alteplase for acute ischaemic stroke: A randomised controlled trial. Lancet.

[25] Broeg-Morvay A, Mordasini P, Slezak A, Liesirova K, Meisterernst J, Schroth G (2017). Does antiplatelet therapy during bridging thrombolysis increase rates of intracerebral hemorrhage in stroke patients?. PLoS One.

[26] Sallustio F, Saia V, Marrama F, Pracucci G, Gandini R, Koch G, et al. Mechanical Thrombectomy for Acute Intracranial Carotid Occlusion with Patent Intracranial Arteries: The Italian Registry of Endovascular Treatment in Acute Stroke. Clin Neuroradiol. 2020; did:21–9. 10.1007/s00062-020-00980-5 33301052

[27] Rangaraju S, Frankel M, Jovin TG (2015). Prognostic Value of the 24-Hour Neurological Examination in Anterior Circulation Ischemic Stroke: A post hoc Analysis of Two Randomized Controlled Stroke Trials. Interv Neurol.

[28] Saver JL, Altman H (2012). Relationship between neurologic deficit severity and final functional outcome shifts and strengthens during first hours after onset. Stroke.

[29] Fleming TR, DeMets DL (1996). Surrogate End Points in Clinical Trials: Are We Being Misled?. Ann Intern Med.

[30] Merlino G, Sponza M, Gigli G, Lorenzut S, Vit A, Gavrilovic V (2018). Prior Use of Antiplatelet Therapy and Outcomes after Endovascular Therapy in Acute Ischemic Stroke Due to Large Vessel Occlusion: A Single-Center Experience. J Clin Med.

[31] Tziomalos K (2014). Type 2 diabetes is associated with a worse functional outcome of ischemic stroke. World J Diabetes.

[32] Tuttolomondo A, Pinto A, Salemi G, Di Raimondo D, Di Sciacca R, Fernandez P (2008). Diabetic and non-diabetic subjects with ischemic stroke: Differences, subtype distribution and outcome. Nutr Metab Cardiovasc Dis.

[33] Spratt N, Wang Y, Levi C, Ng K, Evans M, Fisher J (2003). A prospective study of predictors of prolonged hospital stay and disability after stroke. J Clin Neurosci.

[34] Arboix A, Rivas A, García-Eroles L, de Marcos L, Massons J, Oliveres M (2005). Cerebral infarction in diabetes: Clinical pattern, stroke subtypes, and predictors of in-hospital mortality. BMC Neurol.

[35] Kwak HS, Hwang SB, Jin GY, Hippe DS, Chung GH (2013). Predictors of Functional Outcome after Emergency Carotid Artery Stenting and Intra-Arterial Thrombolysis for Treatment of Acute Stroke Associated with Obstruction of the Proximal Internal Carotid Artery and Tandem Downstream Occlusion. Am J Neuroradiol.

[36] Nedeltchev K, Brekenfeld C, Remonda L, Ozdoba C, Do D, Do, Arnold M (2005). Internal carotid artery stent implantation in 25 patients with acute stroke: Preliminary results. Radiology.

[37] Mpotsaris A, Kabbasch C, Borggrefe J, Gontu V, Soderman M (2017). Stenting of the cervical internal carotid artery in acute stroke management: The Karolinska experience. Interv Neuroradiol.

